# Utilization of miRNAs as Biomarkers for the Diagnosis, Prognosis, and Metastasis in Gynecological Malignancies

**DOI:** 10.3390/ijms252111703

**Published:** 2024-10-31

**Authors:** Alexandros Lazaridis, Hector Katifelis, Emmanouil Kalampokas, Dimitra Lambropoulou, Gerasimos Aravantinos, Maria Gazouli, Nikos F. Vlahos

**Affiliations:** 1 2nd Department of Obstetrics and Gynecology, National and Kapodistrian University of Athens, Vasilissis Sofias 76, 11528 Athens, Greece; dr.alexlazaridis@gmail.com (A.L.); m.kalampokas@gmail.com (E.K.); nfvlahos@gmail.com (N.F.V.); 2 Laboratory of Biology, Department of Basic Medical Sciences, Medical School, National and Kapodistrian University of Athens, Michalakopoulou 176, 11527 Athens, Greece; katifel@med.uoa.gr; 3 ECONCARE, Chatzigianni Mexi 5, 11528 Athens, Greece; d_lampropoulou@yahoo.gr; 4 Oncology Department, Euroclinic Athens, Athanasiadou 7-9, 11521 Athens, Greece; garavantinos@yahoo.gr

**Keywords:** miRNAs, diagnostic biomarkers, metastatic biomarkers, endometrial cancer, vaginal cancer, ovarian cancer, vulvar cancer

## Abstract

Gynecological cancer is a term referring to malignancies that typically involve ovarian, cervical, uterine, vaginal, and vulvar cancer. Combined, these cancers represent major causes of morbidity and mortality in women with a heavy socioeconomic impact. MiRNAs are small non-coding RNAs that are intensively studied in the field of cancer and changes in them have been linked to a variety of processes involved in cancer that range from tumorigenesis to prognosis and metastatic potential. This review aims to summarize the existing literature that has linked miRNAs with each of the female malignancies as potential biomarkers in diagnosis (circulating miRNAs), in tumor histology and prognosis (as tissue biomarkers), and for local (lymph node) and distant metastatic disease.

## 1. Introduction

Gynecological cancer is the term that refers to the malignancies of a woman’s reproductive organs mainly involving ovarian, cervical, uterine, vaginal, and vulvar cancer. Collectively, gynecological malignancies represent increasing trends in morbidity and mortality [1], while it is estimated that gynecological cancers pose a major economic burden that exceeds 80 billion dollars in the USA with extensive inpatient stays and outpatient reviews contributing to an average cost of USD 6300 [2], thus making ovarian cancer the costliest gynecological malignancy when compared to endometrial or cervical malignancies. Antineoplastic hormone agents, sex hormones, and analgesics constituted the highest portion of the prescribed medicines budget. On a more global scale, Sargazi in Iran [3] concluded that ovarian cancer and metastatic cervical cancer contribute to significant financial expenses on the healthcare system. Even though such studies are limited in Europe, there is a recent trend of increasing direct costs due to resource utilization for treatments and follow-ups [4], while the cost increased with disease severity and metastatic status, with an average cost in Spain of EUR 24.000 and EUR 42.000 in stage IV patients, respectively [5]. With regards to African states where the healthcare systems are mostly absent in sub-Saharan territories [6], patients must pay out-of-pocket as highlighted by Dan et al. for cervical cancer patients in Uganda [7].

Biomarkers are molecules that can be quantified in an accurate and producible manner that indicates a particular medical state [8]. They have been widely studied and utilized with the purpose of achieving early diagnosis and improving the overall management of the cancer patient [9]. Among the different types of biomarkers, RNA-based biomarkers offer important advantages when compared to DNA since their dynamic nature favors monitoring and assessment of a patient’s response to treatment and a tumor’s transcription profile, and can also aid in histological classification [10,11]. Currently, there is a vast body of literature concerning the use of RNA-based biomarkers in the fields of diagnosis, treatment response, and follow-up of a variety of pre-malignant and malignant conditions. Micro-RNA (miRNA) biomarkers have been extensively researched in recent years and the fruitful outcomes are promising for the majority of known cancers [12]. Moreover, the number of miRNAs with promising applications is ever expanding not only through standard wet lab approaches including quantitative reverse transcription polymerase chain reaction (qRT-PCR) but also the more sophisticated in silico analyses using Artificial Intelligence (AI) [13]. The aim of this review is to summarize the most recent studies of RNA biomarkers regarding their roles in diagnosis, prognosis, histological classification, and their potential as metastatic disease indicators (both local and distal) in gynecological cancers. Vaginal cancer is not included due to the complete lack of experimental data.

## 2. The Importance of miRNA-Based Biomarkers

MiRNAs represent a type of non-coding RNA that was discovered in 1993. Since then, the study of miRNAs has been intense, revealing important roles both in physiological and in pathophysiological cellular functions [14]. The mechanism of action of these (approximately) 22-nucleotide long RNA molecules concerns the post-transcriptional levels typically resulting in the suppression of their target messenger RNA (mRNA) molecules [15]. Dysregulation of miRNAs is a pivotal step in the majority of human malignancies playing a role in metastatic processes and responses to treatment as revealed in multiple studies [16]. Due to their central role in regulating pre-malignant and malignant pathways, some miRNAs have been described as onco-miRNAs if they have tumor-promoting effects or tumor-suppressor miRNAs if they inhibit tumor-derived mRNAS [17]. The presence of miRNAs in tissue and every biological fluid (including blood and urine), makes these molecules a source of invaluable biomarkers that could assist in early cancer diagnosis and detection of recurrence without the need for invasive procedures [18]. Importantly, RNA detection techniques are becoming extremely sensitive with detection limits as low as three copies [19]. Thus, offering an important advantage of circulating miRNA detection even in cases of extremely limited amounts of tissue available following a directed biopsy. These techniques mainly include qRT-PCR, sequencing techniques (mainly Next-Generation Sequencing, NGS), and nucleic acid sequence-based amplification (NASBA), the latter not widely adopted yet, although it offers an appealing alternative [20,21]. Each detection method has its own strengths and limitations. In particular, q-RT-PCR is widely available and more cost-effective compared to sequencing techniques, which allows not only the detection but also reveals the whole sequence of the RNA molecule of interest [22,23].

## 3. Ovarian Cancer

Ovarian cancer (OC) represents the fifth most common cause of cancer-related death in women globally [24]. Its poor prognosis is multifactorial, being partly attributed to the lack of specific symptoms at the early stages of the disease and the scarcity of reliable biomarkers. Moreover, there is growing evidence that chemoresistance plays a role in reduced therapeutic effects through many different pathways, such as the upregulation of the SLCzA11 (Solute Carrier Family 7 Member 11, also known as xCT) [25] or the activation of tumor-associated macrophages (TAMs), through their immunosuppressive M2-like phenotype that aids in peritoneal metastasis of ovarian cancer cells [26]. Currently, the best available serum biomarker is CA125, which, however, suffers from low specificity [27]. Thus, it is necessary to develop biomarkers that will allow its detection at an early stage, facilitating a more favorable prognosis. Studies regarding MiRNA detection in serum have shown promising results as a diagnostic means of early OC detection. Moreover, the histologic heterogeneity of OC as well as the early identification and localization of metastases represents another diagnostic challenge [28]. Therefore, the identification of biomarkers that could aid the histological classification and others indicative of metastatic disease would offer significant assistance to efficient patient management.

### 3.1. Peripheral Blood miRNAs for Ovarian Cancer Diagnosis

Dysregulated expression of miRNAs is common in OC and many studies have been performed to facilitate an early diagnosis and achieve a more favorable prognosis. A 2023 meta-analysis evaluated the majority of the suggested miRNAs for OC diagnosis and recognized nine upregulated miRNAs (miR-21, miR-125, miR-141, miR-145, miR-205, miR-328, miR-200a, miR-200b, and miR-200c) in the plasma/serum of OC patients [29], while miR-221-3p was separately reported to be increased in OC patient serum [30], and its baseline levels have been proposed as a biomarker of chemotherapy response in colorectal cancer with metastases [31].

Another study by Niemira et al. [32] has proposed a diagnostic model that uses the serum levels of mir-1246 and 150-5p with the aim of diagnosing High-Grade Serous Ovarian Cancer (HGSOC), the most frequent form of OC. Unregulated MiR-1246 serum levels were evaluated for cancer screening and their value was confirmed (especially in breast cancer) in a 2023 meta-analysis [33]. In a similar manner, serum miR-150-5p is also valuable in cancer screening as in the case of renal cancer where it is used as a part of a diagnostic signature that utilizes the expression of four miRNAs (including miR-150-5p) [34].

Downregulation of miRNAs in OC patient blood has also been observed. MiR-26b [35] was found to be reduced in patient serum. Moreover, reduced expression of miRNAs has also been observed in three more miRNAs (miR-34a, let-7f, and miR-31) that collectively form a diagnostic signature for the diagnosis of OC [36].

Utilization of exosome-derived miRNAs has shown promise too as far as the identification of potential diagnostic biomarkers is concerned. Upregulation of five miRNAs (miR-200c-3p, miR-127-3p, miR-346, miR-143-3p, and miR-205-5p) has been reported in exosomes that derive from serum in patients with OC compared to healthy individuals [37]. MiR-200c-3p has been suggested to be of diagnostic significance in the blood of breast [38] and gastric cancer patients [39]. Moreover, miR-200-3p holds an oncogenic role in HGSOC by downregulating the tumor-suppressive gene DLC1 [40]. Similarly, miR-205-5 serum levels have been found to increase in the blood of lung cancer patients [41], while exosome-derived miR-205-5 seems to promote metastasis and angiogenesis by targeting desmocollin-2 [42]. In another study [43], serum exosome miR-34a has shown promise as a diagnostic biomarker for epithelial OC. Upregulation of plasma exosomes of miR-205-5p, miR-145-5p, miR-10a-5p, miR-346, and miR-328-3p was also reported, highlighting the capabilities of exosome-derived miRNAs as a source of diagnostic biomarkers [44].

Interestingly, the utilization of AI as an effort to mine cancer biomarkers has also resulted in the identification of several miRNAs of diagnostic importance. Quite recently, Hamidi et al. [45], identified 10 differentially expressed miRNAs: miR-5100, miR-1290, miR-320b, miR-1233-5p, miR-4783-3p, miR-4532, and miR-4782-3p were upregulated while miR-6800-5p, miR-3184 and miR-1228-5p were downregulated in the serum of OC patient when compared with healthy individuals. It should be noted that upregulated mir-1290 (exosome-derived) has already been suggested as a biomarker for the differentiation of OC from benign neoplasms [46]. In a 2023 study [47] using the random forest algorithm, an additional 10 miRNAs (miR-1290, miR-1233-5p, miR-1914-5p, miR-1469, miR-4675, miR-1228-5p, miR-3184-5p, miR-6784-5p, miR-6800-5p, and miR-5100) were found to be differentially expressed between serum of OC and healthy individuals. Moreover, circulating levels of these miRNAs have been already linked with the diagnosis of malignancies as in the case of serum miR-1290 for lung adenocarcinoma [48].

To further increase the diagnostic efficacy, some studies have investigated the use of miRNA expression in the form of a molecular signature combined with the gold standard biomarker CA125. In epithelial OC, the diagnostic sensitivity of serum miR-125b combined with quantification of serum CA125 levels reached a sensitivity of 95.6% [49]. In a similar approach, the upregulation of serum exosome miR-375 and miR-1307 combined with the evaluation of CA125 has been reported to increase the diagnostic power of CA125 [50]. Finally, by a combinational evaluation of miR-193a-5p along with HE4 and CA125, an efficient diagnostic signature for OC using logistic regression analysis was created and described [51].

The advent of AI has expanded well beyond the initial diagnosis and can offer valuable insights regarding the detection of recurrence. For instance, Aghayousefi et al. [52] developed a diagnostic panel composed of miRNAs with the aim of detecting a possible OC recurrence using a Deep Learning (DL) model. Interestingly, several microRNA-based Machine Learning (ML) models are constantly being developed holding the promise of increasing the diagnostic accuracy and reducing the need for invasive diagnostic means. This crucial process serves as proof that AI will gradually transform the entirety of healthcare [53]. At the same time, there is an increasing number of studies in the literature using AI, which is driving forward the field of cancer miRNAs not only in the field of mRNA biomarkers but also in the field of miRNA–mRNAs interaction, thus potentially revealing unknown mechanisms utilized in cancer management [54].

### 3.2. Ovarian Cancer Tissue Biomarkers for Tumor Histology and Prognosis

Serous ovarian cancer is the most common type of OC accounting for up to 75% of all OC cases [55]. Mucinous Ovarian Carcinoma (MOC) is another type of OC, accounting for less than 5% of all OC cases with an 11% 5-year survival prognosis at advanced stages. However, the histological characterization can be challenging due to the tumor’s increased heterogeneity. Using miRNA tissue biomarkers could aid this process, allowing for efficient management and improved outcomes in these patient groups. In research conducted by Prahm et al., MiR-130a was linked to serous histologic subtype along with advanced FIGO stages. In the same study, miR-34a, miR-455-3p, miR-595, miR-1301, miR-146-5p, 193a-5p, and miR-939 have been shown to be associated with the disease’s clinicopathological features (FIGO Stage and Grade) [56]. Overexpression of miR-153 and miR-485-5p has been proposed as tissue biomarkers that can be used in the discrimination of MOC from serous and endometrioid carcinomas [57]. More recently, Dolivet et al. [58], used pairs of miRNAs as signatures that could distinguish borderline and malignant mucinous ovarian tumors. The 14 pairs used included the ratios of miR-210-3p with miR-195-5p, 152-3p, 199a-3p, 487b-3p, 497-5p, 130a-3p, 26a-5p, 195-5p, 152-3p, 487b-3p, 497-5p, 199a-3p, 130a-3p, and 26a-5p. Another study has also recognized the upregulation of miR-192, miR-194, and miR-215 in MOC, which could also be of diagnostic importance [59]. Ovarian Clear Cell Carcinoma (OCCC) is another rare type of EOC characterized by its poor prognosis and its chemotherapy resistance phenotype [60]. Currently, miR-510 has been found in increased levels, compared to HGSOC tissues, making miR-510 a potential diagnostic candidate that could assist with histological characterization [61]. In colon cancer, miR-510 is an independent prognostic factor where its increased levels have been associated with advanced TNM stage, invasion, and poor tumor differentiation [62]. A considerable amount of research has been performed on the identification of miRNAs with prognostic importance. Upregulation of miR-1908 in OC tissue has been proposed to indicate a favorable prognosis, while reduced miR-1908 expression results in decreased disease-free and survival time [63]. Upregulation of another miRNA, let-7a, indicates a favorable outcome in patients treated with cisplatin, who have a lower risk of death and disease progression [64]. On the contrary, upregulation of miR-616 serum has been shown to confer unfavorable clinical outcomes. Increased miR-616 levels have been correlated with poor differentiation and a decrease in both overall and disease-free survival [65]. The suggested mechanism involves the reduction in E-cadherin and N-cadherin, which is attributed to the upregulation of mir-616. Similarly, patients with increased miR-196b expression were also shown to have a poor prognosis [66], and its overexpression has also been found to promote invasion in recurrent OC [67]. Downregulated miR-494 has been associated with unfavorable prognostic features that include tumor size and FIGO stage [68]. Similarly, reduced levels of miR-510 and 129-3p serve as a poor prognosis indicator. Additionally, a decrease in miR-510 has been associated with early relapse [61]. Low levels of miR-509-5p have been shown to pose prognostic significance in OC patients and indicate reduced overall survival [69].

### 3.3. miRNAs as Indicators of Metastatic Disease in Ovarian Cancer

Lymph node metastasis (LNM) is frequently observed in OC, with studies reporting more than 50% of patients positive for LNM being in advanced OC [70]. Biomarkers that could indicate the possibility of LNM involvement would be invaluable. Currently, several miRNAs have been proposed for that purpose. Upregulated miR-18b [71], miR-19b [72], and miR-216a [73] have been associated with LNM in OC tissues. The involved mechanism of action typically affects signaling pathways, as in the case of mir-19-b, which inhibits the PTEN/AKT pathway [72]. Reduced tumor tissue expression of miR-193b [74] has been correlated with both ascites and LNM. A reduction in miR-193b is considered to indicate LNM and distant metastasis in several cancers, as suggested by a meta-analysis conducted by Yu et al. [75]. Similarly, reduced miR-100 expression was also correlated with LNM [76]. MiR-100 remains less studied in the context of LNM and distant metastases in humans; however, it has been found to promote metastasis in a mouse model via the STAT5A/IL-1RA pathway [77]. Studies have also revealed a link between miRNA expression in OC tissue and distant metastasis. Decreased miR-489 [78] and miR-532-5p [79] have been associated with distant metastases, while decreased miR-26b levels have been associated with both distant metastases and recurrence [80]. A reduction in miR-206 [81] has been correlated with both LNM and/or distant metastases. Although not studied in OC, a key effect of miR-206 is the downregulation of TM4SF1, which regulates cell growth and activation [82].

Regarding serum miRNA expression and association with metastatic disease, there are very few reports in the literature. Reduced mir-125b expression was further observed in the serum of patients with LNM and/or distant metastatic disease [83]. Similarly, decreased levels of miR-199a [84] were also correlated with LNM and/or distant metastatic disease. On the contrary, three miRNAs (miR-200c-3p, miR-346, and miR-127-3) were elevated in the serum of patients exhibiting metastatic disease compared to OC patients without [44]. It should be noted that these miRNAs have been investigated for their pathogenetic role in tumorigenesis, but outside of OC, they have not been used as serum biomarkers in cancer with few exceptions as in the case miR-125b, which has been suggested to hold promise as a biomarker with prognostic significance in lung cancer [85].

## 4. Endometrial Cancer

Endometrial cancer (EC) is the second most common gynecological malignancy and the recent reported incidence of 15 to 25 per 100,000 women in Western countries is rising (25% increase over the last decade) [86]. Endometrial cancer is a heterogeneous malignancy comprising many histological types, which differ significantly in terms of pathogenesis, clinical presentation, and prognosis. Such heterogeneity impedes the development of screening and treatment strategies. The condition is grossly associated with an increase in the prevalence of risk factors, mainly obesity, nulliparity, and an aging population. Approximately a third of endometrial cancers are secondary to lifestyle and additional external factors, a fact that grants them to be potentially preventable [87,88]. Low-grade endometrial cancers (type 1) are typically identified at an early stage, a fact that results in improved survivorship. More aggressive (type 2) endometrial malignancies (serous, clear cell), although less common than their endometrioid endometrial carcinoma (EEC) counterpart, are responsible for a disproportionate number of endometrial cancer-related deaths [89].

### 4.1. Peripheral Blood miRNAs for Endometrial Cancer Diagnosis

Benati et al. reviewed 75 patients, including 45 women with endometrial cancer, and identified higher circulating miR-203 levels in EC patients compared to controls. Moreover, aberrant miR-203 methylation was assessed but did not seem to affect cancer biology [89]. Fan et al. [90] described two miRNA signatures following a study protocol composed of four phases, whereby they evaluated patients with EC who were diagnosed via histological examination but declined any therapeutic intervention such as surgery, chemotherapy, or radiotherapy prior to sampling. They then reported on a three-miRNA signature (miR-142-3p, miR-146a-5p, and miR-151a-5p), which showed an increased expression in the plasma of EC patients (compared with healthy individuals). Interestingly, according to the analysis of FIGO stages, no statistically significant difference was observed regarding the expression levels of the miRNAs during early stages (90% of samples) and advanced stages (remaining 10% of samples) [90]. Moreover, in a prospective study where 92 patients diagnosed with EC and 102 healthy female individuals were enrolled, a six-miRNA signature (miR-143-3p, miR-195-5p, miR-20b-5p, miR-204-5p, miR-423-3p, and miR-484) was proposed. It was significantly overexpressed in the serum of EC compared with normal female controls [91]. Oropeza-de Lara et al. in their review studied 138 miRNAs with potential diagnostic, prognostic, or treatment response potential in EC. They showed that seven diagnostic panels (in serum, in plasma, and in endometrial tissue) have improved sensitivity and specificity for EC diagnosis compared to individual miRNAs [92]. Torres et al. [93] described that tissue miRNA signatures were independent prognostic markers of overall and progression-free survival (miR-1228/miR-200c/miR-429). Analysis using Logistic regression analysis revealed two miRNA-based signatures: miR-92a/miR-410 and miR-92a/miR-205/miR-410, which were capable of classifying tumor tissues with significantly improved accuracy when compared to single miRNAs. Furthermore, miRNA signatures in plasma samples of miR-9/miR-1228 and miR-9/miR-92a classified EEC samples showing high accuracy.

### 4.2. Endometrial Cancer Tissue Biomarkers for Tumor Histology and Prognosis

Over the last few decades, the role of miRNA expression in endometrial cancer has been evaluated and the pathways of dysregulated expression have been reviewed. Specific miRNAs are expressed in various tissues and changes in regulation of gene expression are thought to cause carcinogenesis. Thus, tissue-specific miRNAs may serve as biomarkers for EC diagnosis and prognosis [94]. Donkers et al. reviewed 26 studies (1400 EC patients) that reported on 106 differentially expressed miRNAs. The most commonly found miRNA that showed upregulation was miR-205, which was followed by the miR-200 family (miR-200c, miR-200a, miR-200b, miR-429, and miR-141) and miR-223, miR-182, and miR-183. In addition, miR-135b was also frequently upregulated. Nevertheless, so far there has been no robust consensus on downregulated miRNAs [95]. Kalinkova et al. [96] evaluated differences in miRNA expression between well- and poorly differentiated (grades 1 and 3) endometrioid endometrial carcinomas (EEC), as well as between EEC and serous endometrial carcinoma (SEC). Poorly differentiated cancers exhibited lower expressions of let-7c-5p, miR-125b-5p, miR-23b-3p, and miR-99a-5p as compared to well-differentiated tumors. Regarding SEC, let-7g-5p, miR-195-5p, miR-34a-5p, and miR-497-5p were significantly downregulated. Moreover, the downregulation of miR-497-5p was prominent in SEC, clear cell carcinomas (CCCs), and carcinosarcomas (CaSas) compared to EECs. It is proposed that miRNAs influence crucial EC-related molecular pathways such as the JAK/STAT axis, EGFR, TGF-β signaling, and P53 [97]. Cohn et al. [98] performed miRNA extraction from the histological samples of 141 women with EC and reported that the miRNA profile is distinct from a normal endometrium, even in patients with early-stage tumors (IA grade 1). Klicka et al. [99] investigated the expression levels and the roles of 106 miRNAs in the invasion and the metastatic potential of EC. The study concluded that 63 of the samples acted as tumor suppressor miRNAs, and 38 were oncomiRNAs.

### 4.3. miRNAs as Indicators of Metastatic Disease in Endometrial Cancer

The upregulation of the miR-200 family [100] and miR-205 [101] in endometrial cancer compared to controls is well reviewed and thought to be implicated in the epithelial-to-mesenchymal transition, tumor invasion, and metastasis [102]. In particular, the miR-200 family consisting of five miRNAs (miR-200a, miR-200b, miR-200c, miR-141, and miR-429) negatively regulate the expression of ZEB-1/2, which plays a critical role in epithelial-to-mesenchymal transition [103]. In EC, this biological process is particularly important because myometrial invasion is one of the most important prognostic factors for the risk of disease spread outside the uterus, especially lymph node metastasis. Lee et al. [100] identified that miR-200s are highly expressed in EECs compared with that of normal endometrial tissues and when they treated endometrial cancer cells with specific anti-miRNAs, the cytotoxic activity of chemotherapeutic agents was enhanced. The miRNAs that are overexpressed in stage I endometrioid endometrial cancers (such as mir-200c and mir-183) will target and eventually downregulate the PTEN (Phosphatase and tension homolog) gene, which is one of the most inactivated tumor suppressor genes in sporadic cancers; altered in 40–60% of endometrial cancers. Furthermore, miR-182 and miR-183 seem to downregulate the expression of the tumor-suppressor gene FOXO1 in endometrial cancer [104]. Individual miRNAs may regulate multiple malignancies, such as miR-205 and miR-203, that are also expressed in ovarian and renal tract cancers [105]. Analyses employing immunohistochemistry of the tissue microarrays that derive from these patients have established the functional connection among the reduced levels of tumor-suppressive miRNAs and their target oncogenes (namely, *ERBB2*, *EGFR*, *EPHA2*, *BAX*, *GNA12*, *GNA13*, and *JUN*). Finally, the tumor-suppressive miR-142 cluster and miR-15a may serve as prognostic molecules for therapy response [106].

## 5. Cervical Cancer

Cervical cancer represents the fourth most frequent cancer in women worldwide [107] and one the most extensively studied malignancies due to the understanding of the progression from the Human Papillomavirus (HPV) persistent infection to the three degrees of cervical intraepithelial neoplasia (CIN 1-3), which are precursor pre-cancerous lesions and, eventually, over a period, lead to invasive cancer [108]. Through complex genetic and epigenetic pathways (including miRNA involvement), the cell cycle control pathways are dysregulated by increased expression of the HPV E6 and E7 proteins which, among other functions, are capable of deactivating the products of the RB and p53 immunosuppressive genes [109].

### 5.1. Peripheral Blood miRNAs for Cervical Cancer Diagnosis

A recent systematic review evaluated the expression of 33 microRNAs, 17 of these molecules (namely, miR-9, miR-15b, miR-16-2, miR-21,miR-34a, miR-92a, miR-152, miR-155-5p, miR-192, miR-196a, miR-199a-5p, miR-205, miR-218, miR-425-5p, miR-497, miR-1266, and miR-1290) were upregulated in women with precursor lesions and cervical cancer while 16 microRNAs were downregulated compared to healthy individuals (let-7d-3p, miR-30d-5p, miR-34a, miR-100, miR-125b, miR-145, miR-195, miR-200a, miR-203a-3p, miR-214, miR-218, miR-370, miR-521, miR-638, miR-1914-5p, miR-2861) [110]. The importance of developing a standardized approach was highlighted by employing rigorous sampling including HPV testing in every sample and a universal quantitative evaluation of microRNA expression to radically reduce any methodological pitfall. In a separate study, the random forest algorithm identified the top eight miRNAs (let-7a-3p, let-7d-3p, miRNA-30d-5p, miRNA- 144-5p, miRNA-182-5p, miRNA-183-5p, miRNA-215-5p and miRNA-4443) from an initial pool of 37 differentially expressed miRNAs (DEmiRs), and it had the highest clinical prediction accuracy [111]. Additionally, miR-30d-5p and let-7d-3p can serve as a non-invasive screening for cervical cancer and its precursor conditions. Interestingly, miRNA-125a-5p represents a prime candidate of a potential biomarker capable of differentiating between non-cervical and cervical cancer [112]. Table 1 summarizes the main miRNAs that have been suggested in the literature for the diagnosis of the gynecological malignancies that are discussed in this review.

### 5.2. Cervical Cancer Tissue Biomarkers for Tumor Histology and Prognosis

Considerably high variability in tissue miRNA expression has been observed, most notably among normal cervical samples. Nevertheless, once this variability is accounted for, deregulated miRNAs can be found in both malignant and pre-malignant cervical tissues. In a microarray study, Pereira et al. [115] investigated the expression of eight miRNAs (miR-26a, miR-29a, miR-99a miR-143, miR-145, miR-199a, miR-203, and miR-513) that were gradually reduced alongside five miRNAs (miR-10a, miR-132, miR-148a, miR-196a, and miR-302b) that gradually upregulated in CIN1, CIN3, and squamous cell carcinoma (SCC) samples, respectively. Additionally, six miRNAs (miR-16, miR-27a, miR-106a, miR-142-5p, miR-197, and miR-205) were found to decrease from normal tissue to CIN, then increase from CIN to SCC. Conversely, miR-522-5p and miR-512-3p showed the opposite trend, with an initial upregulation that was followed by a downregulation [116]. Among the downregulated miRNAs, miR-34a, miR-125, and miR-375 were found to be progressively dysregulated when moving from normal epithelium to ICC [117]. Additionally, miR-125b is implicated in HPV-induced carcinogenesis in two distinct pathways: a) HPV-L2 homology necessary for the viral capsid assembly; and b) miR-125b mediated 53-pathway inactivation, thus maintaining cells viable with viral genomes inside [118]. In a subsequent study [119] that included HPV16-positive SCCs and CIN samples, Li Y and colleagues reported on 31 miRNAs that revealed important trends from unaffected/normal epithelium to cancer (14 downregulated and 17 upregulated). Six miRNAs (miR-29a, miR-92a, miR-99a, miR-155, miR-195, and miR-375) were validated and were subsequently confirmed by qPCR in 91 biopsies. Importantly, the downregulation of miR-99a and miR-29a was in accordance with the results of Pereira et al. [115]. Another study [118] also found several miRNAs dysregulated (an upregulation of 16 miRNAs and a downregulation of 10 more miRNAs among SCC and normal tissue). MiR-21, miR-21-3p, miR-15b, and miR-16 were the most upregulated while miR-376 and miR-218 showed the sharpest downregulation. MiR-218 plays an important role in the regulation of the epigenetic machinery in cervical cancer. It targets a plethora of genes, *LAMB3* included, which is known to promote cell migration and carcinogenesis not only in mice models but also in human keratinocytes [120]. MiR-21 expression is upregulated with neoplasms of many anatomic regions that include cervical pre-malignant lesions and intrahepatic cholangiocarcinoma (ICC) [121]. MiR-218 was downregulated by High-Risk-HPV (HR-HPV) in infected sites (more in CIN2/3 compared to CIN1) [122]. Increasing miR-21 expression has been linked to disease progression. Disturbed levels of miR-21 and let-7a are highly associated with aberrant expression and activation of STAT3 and seem to be tightly linked with HPV16 infection [122]. Additionally, let-7c expression is gradually downregulated in CIN of increasing grade and its expression is likely induced as a response to the activation of p53 [123]. Another study [124] reported more than 106 miRNAs to be differentially regulated in CIN2/3 and invasive cervical cancer (SCC) compared to normal epithelium. In the same study three different categories were identified, including early continuous (molecules that exhibit concordant differential expression), early transient (differential expression of only CIN2/3 compared with normal), and late miRNAs (differential expression of only SCC compared with normal specimens). Additional important miRNAs include miR-9 and miR-100. The downregulation of the latter seems to contribute to cervical carcinogenesis by promoting cell growth and via the acceleration of the G2/M phase of the cell cycle while at the same time it could decrease apoptosis [125]. MiR-9 is upregulated in several cancers and its expression increases more in SCC than in premalignant stages (CIN2-3). Interestingly, miR-9 stimulates angiogenesis via downregulating E-cadherin, which has been found to activate β-catenin, and ultimately resulting in the increased expression of *VEGFA*, a potent pro-angiogenic factor [126]. MiR-34a is considered a tumor-suppressive miRNA in HPV-induced cervical transformation. Gocze et al. [127] found that p53 regulates miR-34a and that the HR-HPV E6 it induces is capable of inhibiting it via p53. Consequently, females with a normal cervix and who have an HPV infection show increased levels of miR-34a, and the expression further increases during cancer cases [128].

### 5.3. miRNAs as Indicators of Metastatic Disease in Cervical Cancer

Current evidence [129] shows that circulating miR-20a and miR-203 are highly increased in cervical cancer patients when compared to healthy individuals. Circulating miR-20a appears as a valuable biomarker for the differentiation of positive lymph node metastasis. Ma et al. [130] reported that miR-205 in serum was increased in cervical cancer patients compared with healthy individuals. Increased levels of miR-205 showed a correlation with poor tumor differentiation, lymph node metastasis, and increased tumor stage, thus potentially serving as a predictive quantitative biomarker regarding the prognosis of cervical cancer patients. Additionally, circulating miRNA-218 [131] and miRNA-1290 [132] may be of use for cancer staging and metastases in lymph nodes, since their increased serum levels correlate with advanced stage. MiR-21 reduces the expression of RASA1 in cervical cancer cell lines. The Ras-induced epithelial–mesenchymal transition contributes to the miR-21/RASA1 axis that promotes the migration of cancer cells. The latter highlights the importance of miR-21 as a potential biomarker in the detection of lymph node metastasis [133]. Table 2 summarizes the main miRNAs used in the detection of metastatic disease for all gynecological malignancies described in this review.

## 6. Vulvar Cancer

Vulvar cancer is a rather rare gynecological malignancy that accounts for approximately 4% of all gynecological malignancies. Currently, there are no specific screenings for this cancer, and patients commonly first present due to chronic vulval itching, irritation, and pain with or without a vulval lump, lesion, or ulceration [132]. Five-year survival is high in stage I (93–100%) but is radically reduced in more advanced stages [133,137]. Up to now, there are very few studies that have investigated the use of miRNAs in the fields of diagnosis, prognosis, and metastasis of vulvar cancers. This observation is possibly due to the infrequency of these malignancies and highlights the need for further research on miRNA biomarkers that could facilitate its management.

### 6.1. Peripheral Blood miRNAs for Vulvar Cancer Diagnosis

Currently, only one study, by Bujko et al., is available in the literature regarding the role of peripheral blood miRNAs in discriminating pre-cancerous vulvar lesions (High-grade squamous intraepithelial lesions, HSIL, and differentiated vulvar intraepithelial neoplasia, dVIN) from cancer (vulvar squamous cell carcinoma, VSCC) [138]. In this study, upregulation of circulating miR-431-5p has been proposed as a diagnostic of VSCC (compared to its expression in pre-cancerous lesions). This miRNA has been found to act as a tumor suppressor in pancreatic cancer [139] and to promote invasion and proliferation in colon cancer [140]. Of note, miR-431-5p has also been studied as a promising biomarker in non-small cell lung cancer (NSCLC), but there was no statistical significance between patients and healthy individuals [141].

### 6.2. Vulvar Cancer Tissue Biomarkers for Tumor Histology and Prognosis

Dysregulation of a plethora of miRNAs has been evaluated between adjacent non-cancer tissue and vulvar squamous cell carcinoma. Importantly, two miRNAs show upregulation (miR-182-5p and miR-183-5p) while miR-603, miR-107, and miR103a-4p showed decreased expression [142]. Both miRNAs (miR-182-5p and miR-183-5p) are important contributors to cancer development [143,144] and have been associated with poor outcomes in several cancers [145,146]. Regarding miR-603, the evidence suggests its role as a tumor suppressor in cancer, and it can result in poor prognosis due to the upregulation of its target, eEF2K, in breast cancer patients [147]. MiR-107 also acts as a tumor suppressor and its upregulation has been associated with increased stages of colorectal cancer [134]. On the other hand, although miR-103a remains less studied, it seems that it inhibits cell death and boosts glycolysis in liver cancer where it indicates a poor prognosis [135] while it also promotes NSCLC proliferation via the Akt Pathway [148]. Another study [149] showed that the downregulation of both miR30c and let-7a is associated with the increased expression of the HMGA2 gene (High-Mobility Group AT-hook 2). The function of HMGAG2 is critical in epithelial-to-mesenchymal transition and tumorigenesis [136]. It has been associated with poor prognosis in several cancers, including renal cell carcinoma (reduced OS) [150] and in pancreatic cancer [151], but it remains inadequately studied in vulvar cancer prognosis. Finally, upregulation of miR-3147 has been shown to indicate a poor patient outcome via its effects on SMAD4 [152]. Upregulation of miR-3147 has been correlated with increased invasion of VSCC but has not been found to be correlated with tumor size and differentiation LNM or vascular invasion.

### 6.3. miRNAs as Indicators of Metastatic Disease in Vulvar Cancer

Currently, only two miRNAs have been associated with LNM, miR-223-5p and miR-19-b1-5p. For both miRNAs, their reduced tissue expression has been associated with LNM [153]. The effects of the former have been studied throughout different malignancies where evidence suggests that the members of the miR-223 family act as both an oncogene and oncosuppresor in a manner dependent on the involved biological process [154] Reduced expression of miR-223-5p has also been observed in metastatic NSCLC tissues [155], while targeting miR-223-5p has been found to inhibit prostate cell migration [156]. On the contrary, miR-19-b1-5p remains poorly studied, even though the mir-19 family has been shown to promote tumor progression and is of prognostic significance in breast cancer [142]. Similarly, increased levels of mir-590-5p are linked with the presence of LNM in VSCC [157]. With roles that can either promote carcinogenesis or can be tumor-suppressive, miR-590-5p has been characterized as a “double-edge sword” [158]. This can justify contradicting literature where it has not only been found to inhibit stemness and metastatic potential in breast cancer [159] and reduce invasiveness in NSCLC [160] but also to act as an oncogene in bladder [161] and liver malignancies [162]. The connection between miRNAs and vascular invasion has also been investigated [153]. Apart from the correlation between its dysregulated expression and LNM, reduced levels of miR-191-b1-5p and miR-100-3p have been linked with vascular invasion. MiR-100-3p has been suggested to inhibit proliferation and trigger apoptosis in gastric cancer [163] and to play an additional role in cellular differentiation [164].

## 7. Discussion

**Figure 1:**
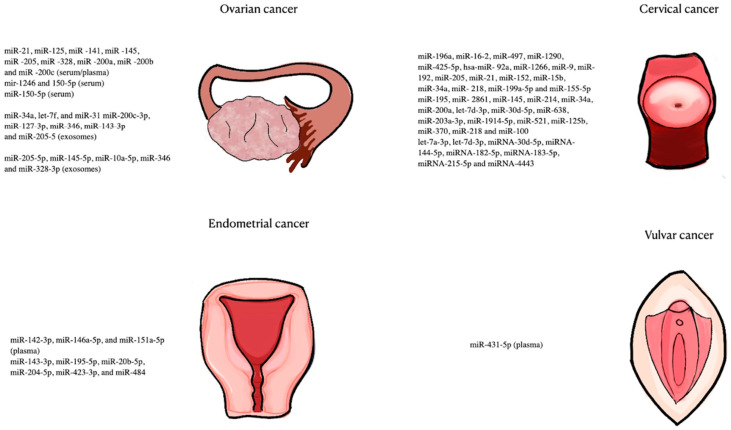
Dysregulated miRNAs in ovarian, cervical, endometrial, and vulvar cancer.

Gynecological malignancies represent the most common types of cancer in females. Oftentimes, diagnosis is set when metastasis is already present, a factor that contributes to increased morbidity and mortality. MicroRNAs provide the unique opportunity to develop novel and adequately sensitive/specific biomarkers that will be beneficial in every aspect of cancer management, ranging from early diagnosis to the identification of metastatic disease. Figure 1 summarizes the dysregulated miRNAs in gynecological cancers, showing a large number of miRNAs with potential as diagnostic biomarkers. In ovarian cancer, miRNAs have been shown to increase the diagnostic efficacy of CA125 when combined together. There is also increasing utilization of miRNAs in lymph node metastasis, which can aid timely staging and follow-up of these patients. In endometrial cancer tissue, miRNAs can potentially differentiate between the different histological grades and different subtypes of endometrial cancer. Moreover, in cervical cancer, HPV triage and liquid-based cytology (smear test) constitute the biggest screening tools utilized as a preventive strategy, but they are both fallible and have their limitations. Understanding the molecular and (epi)genetic pathways linked to the pathogenesis of SCC offers a unique opportunity to control disease through timely diagnosis and treatment. Biomarkers can serve for the early detection of malignant transformations, thus making it possible to curb cancer-related morbidity and mortality in the future. Finally, vaginal cancer is largely unstudied and there are no data available linking it with miRNA biomarkers. Future studies should at least focus on studying miRNAs that have already shown promise in other gynecological malignancies.

## 8. Conclusions

This literature review summarizes the most commonly researched miRNAs as potential biomarkers for the timely diagnosis and future prognosis of the main gynecological malignancies, including ovarian, endometrial, cervical, and vulval cancer, with the exception of vaginal cancer, whereby the literature remains scanty. It is immediately evident that the universal and standardized utilization of these molecular biomarkers will open new horizons in the management of cancer patients. A potential future research goal within grasp would be the development of readily available non-invasive diagnostic kits for the main gynecological cancers.

## Figures and Tables

**Table 1:** List of miRNAs that have been associated with the diagnosis of ovarian, vulvar, endometrial, and cervical cancer.

Malignancy	miRNA	Clinical Use	Reference
Ovarian Cancer	miR-21, miR-125, miR-141, miR-145, miR-205, miR-328, miR-200a, miR-200b, and miR-200c (serum/plasma)	Upregulation can be used for diagnosis	[16]
	mir-1246 and 150-5p (serum)	Diagnostic model for diagnosis	[19]
	miR-150-5p (serum)	Molecular signature for diagnosis	[21]
	miR-34a, let-7f, and miR-31	Molecular signature for diagnosis	[23]
	miR-200c-3p, miR-127-3p, miR-346, miR-143-3p, and miR-205-5 (exosomes)	Upregulation can be used for diagnosis	[24]
	miR-205-5p, miR-145-5p, miR-10a-5p, miR-346, and miR-328-3p (exosomes)	Proposed as diagnostic biomarker	[31]
Vulvar Cancer	miR-431-5p (plasma)	Upregulation for diagnosis	[73]
Endometrial Cancer	miR-142-3p, miR-146a-5p, and miR-151a-5p (plasma)	Molecular signature for diagnosis	[108]
	miR-143-3p, miR-195-5p, miR-20b-5p, miR-204-5p, miR-423-3p, and miR-484	Molecular signature for diagnosis	[109]
Cervical Cancer	miR-196a, miR-16-2, miR-497, miR-1290, miR-425-5p, hsa-miR-92a, miR-1266, miR-9, miR-192, miR-205, miR-21, miR-152, miR-15b, miR-34a, miR-218, miR-199a-5p, and miR-155-5p	Upregulation in cervical cancer and precursor lesions	[113]
	miR-195, miR- 2861, miR-145, miR-214, miR-34a, miR-200a, let-7d-3p, miR-30d-5p, miR-638, miR-203a-3p, miR-1914-5p, miR-521, miR-125b, miR-370, miR-218, and miR-100	Upregulation in cervical cancer and precursor lesions	[113]
	let-7a-3p, let-7d-3p, miRNA-30d-5p, miRNA-144-5p, miRNA-182-5p, miRNA-183-5p, miRNA-215-5p, and miRNA-4443	miRNA panel for diagnosis	[114]

**Table 2:** List of miRNAs that have been associated with the presence of metastatic disease in ovarian, vulvar, endometrial, and cervical cancer. The sample type (tissue or serum) is given in parenthesis. “LNM” represents Lymph Node Metastasis, “DM” represents Distant Metastasis and “VI” represents Vascular Invasion.

Malignancy	miRNA	Expression	Reference
Ovarian Cancer	mir-18b (tissue)	Increased (LNM)	[56]
	miR-19b (tissue)	Increased (LNM)	[57]
	miR-216a (tissue)	Increased (LNM)	[58]
	miR-193b (tissue)	Reduced (LNM)	[59]
	miR-100 (tissue)	Reduced (LNM)	[61]
	miR-489 (tissue)	Reduced (DM)	[63]
	miR-532-5p (tissue)	Reduced (DM)	[64]
	miR-26b (tissue)	Reduced (DM)	[65]
	miR-206 (tissue)	Reduced (LNM, DM)	[66]
	mir-125b (serum)	Reduced (LNM, DM)	[68]
	miR-199a (serum)	Reduced (LNM, DM)	[61]
	miR-200c-3p (serum)	Increased (DM)	[31]
	miR-346 (serum)	Increased (DM)	[31]
	miR-127-3 (serum)	Increased (DM)	[31]
Vulvar Cancer	miR-223-5p (tissue	Reduced (LNM)	[91]
	miR-19-b1-5p (tissue	Reduced (LNM)	[91]
	miR-191-b1-5p (tissue)	Reduced (VI)	[91]
	miR-100-3p (tissue)	Reduced (VI)	[91]
Endometrial Cancer	miR-200 family (tissue)	Increased (DM)	[119]
	miR-205 (tissue)	Increased (DM)	[120]
Cervical Cancer	miR-20a (serum)	Increased (TNM)	[134]
	miR-205 (serum)	Increased (TNM)	[135]
	miR-21 (serum)	Increased (LNM)	[136]

## Data Availability

Not applicable.
